# Prevalence and Associated Factors of Depression, Anxiety and Insomnia Symptoms Among Patients Receiving Ophthalmic Consultation Online During the COVID-2019 Pandemic

**DOI:** 10.3389/fpsyt.2022.855366

**Published:** 2022-03-21

**Authors:** Lin Ding, Ni Guo, Jianing Zhu, Dilinuer Tuxunjiang, Aierxiding Abudoukeremuahong, Chengguo Zuo, Mingkai Lin

**Affiliations:** ^1^Department of Ophthalmology, People's Hospital of Xinjiang Uygur Autonomous Region, Urumqi, China; ^2^State Key Laboratory of Ophthalmology, Guangdong Provincial Key Laboratory of Ophthalmology and Visual Science, Zhongshan Ophthalmic Center, Guangdong Provincial Clinical Research Center for Ocular Diseases, Sun Yat-sen University, Guangzhou, China

**Keywords:** coronavirus disease 2019, ophthalmic online consultation, patients, depression, anxiety, insomnia

## Abstract

**Objective:**

This study aims to assess the prevalence and associated factors of depression, anxiety and insomnia symptoms among patients undergoing ophthalmic consultation online during the coronavirus disease 2019 (COVID-2019) pandemic.

**Methods:**

We reviewed the data of patients who received online ophthalmic consultations during the lockdown period from February to August 2020, and an online survey was conducted among them. We collected the respondents' demographic data and their attitudes toward the online consultation, assessed the depression, anxiety and insomnia symptoms and estimated associated factors by logistic regression analysis.

**Results:**

The online service provided 425 consultations during the COVID-19 lockdown period. Of these eligible subjects, 139 patients responded to an invitation to participant in the study (105/75.5% were females, and 40/28.8% were health care workers). More than half of the participants reported that they trusted and were satisfied with the online consultation (109/78.4% and 82/59%, respectively). Fifty-two (37.4%), 32 (23.0%), and 53 (38.1%) patients showed symptoms of depression, anxiety, and insomnia, respectively. Depression was found to be significantly more common in health care workers (*P* = 0.019) and those who were basically satisfied with online consultation (*P* = 0.024). Anxiety was more common among participants who had used electronics for a long time (*P* = 0.038). Binary logistic regression showed health care work as a risk factor for depressive symptoms (odds ratio [OR]: 2.424; 95% CI: 1.143–5.143; *P* = 0.021).

**Conclusion:**

Psychological distress is highly prevalent among patients who were involved in online consultation for ocular manifestations during the COVID-19 lockdown period. In the context of a major public health event, ophthalmologists should focus not only on ocular symptoms but also on the mental health of their patients, and appropriate psychological support should be given.

## Introduction

Coronavirus Disease 2019 (COVID-19) is a public health emergency of international concern, according to a World Health Organization declaration on January 31, 2020 (1, 2). Since the start of the pandemic, there have been over 370 million reported cases and over 5.6 million deaths globally according to February 1, 2022, data from the WHO (3).

The rapid spread of the disease via close contact between people is an important feature of COVID-19 (4). It has prompted precautions in public places, such as hospitals, which are prone to transmission of communicable diseases due to the large number of people congregating in confined spaces. To curb the spread of the disease, compulsory measures were being implemented by governments, such as social distancing, isolation/lockdowns, and social activity restrictions) (5, 6). These restrictions have brought about great changes in people's life. Firstly, non-COVID-19 patients' medical needs have been greatly affected, which may have caused psychological distress. However, the emergence of a virtual hospital, not limited by place or time that enabled patients to see physicians while avoiding a crowded waiting room, became an alternative for many patients (7). Online consultation can address some patients' needs, but it is uncertain whether seeing a health care provider virtually can affect patients' mental state. Secondly, measures of remote working were also being imposed in many organizations. Coupled with home quarantine, there had been a marked decrease in outdoor activity and an increase in screen time, which had been shown to be associated with mental health problems (8).

Some studies have investigated the psychological impact related to the COVID-19 crisis on people. One online survey of 56,679 individuals from all 34 provincial-level regions in China showed that 27.9% of respondents reported depression, 31.6% anxiety, 29.2% insomnia, and 24.4% acute stress, as evaluated by the Patient Health Questionnaire-9, Generalized Anxiety Disorder-7, Insomnia Severity Index, and Acute Stress Disorder Scale (9). Another survey of medical staff found rates of anxiety and depressive symptoms were 13.3 and 18.4%, respectively (10). However, there are few studies on the psychological status of patients, especially those who consult with ophthalmologists online.

This study collected the consultation information of ophthalmic patients at the “online hospital” and conducted an online psychological questionnaire survey with the aim of assessing ophthalmic patients' symptoms of depression, anxiety, and insomnia, as well as analyzing potential associated factors with these symptoms during COVID-19 lockdown to provide a basis for interventions in public health emergencies.

## Methods

Data (including name, sex, and consultation content) of patients receiving online ophthalmology consultation during the lockdown period (from February 15, 2020 to August 14, 2020) were collected through the online consultation platform of People's Hospital of Xinjiang Uygur Autonomous Region. We divided the patient consultation content into 12 sections: blurred or different vision, eye discomfort or abnormal appearance, fluttering shadows before eyes, myopia and abnormal eye position, outpatient follow-up and further treatment, and abnormal secretions, ocular trauma, eye surgery, postoperative follow-up, invalid consultations such as just saying hello without describing any symptoms or describing conditions that have nothing to do with ophthalmology, repeated consultations, and other such as asking about medication usage, whether medication can be delivered by express delivery, the cost of certain tests and the availability of doctors for work.

The subjects of this study were the aforementioned patients who underwent online ophthalmic consultations during the COVID-19 lockdown. We sent questionnaires online to these people through Wenjuanxing, a survey platform in China. The first part of the online questionnaire recorded basic information about the participants (including name, sex, age, education level, whether they were medical workers, current status, whether they had used electronic products for a long time). The second part of the questionnaire involved the use of three tools to assess patients' symptoms of depression, anxiety, and insomnia, respectively: the Chinese versions of the nine-Item Patient Health Questionnaire (PHQ-9) (11), the seven-item Generalized Anxiety Disorder scale (GAD-7) (12) and the seven-item Insomnia Severity Index (ISI) (13). The last part of the questionnaire surveyed participants' trust in and satisfaction with online counseling. These measurement tools were scored as follows: PHQ-9, normal (0–4), mild (5–9), moderate (10–14), moderately severe (15–19), and severe (20–27) depression; GAD-7, normal (0–4), mild (5–9), moderate (10–14), and severe (15–21) anxiety; ISI, normal (0–7), subthreshold (8–14), moderate (15–21), and severe (22–28) insomnia.

This study was a cross-sectional online survey conducted from August 16 to August 29, 2020, which was approved by the Ethics Committee of Zhongshan Ophthalmic Center, Sun Yat-sen University, and People's Hospital of Xinjiang Uygur Autonomous Region. Written informed consent was received online from all participants before the study.

## Statistical Analysis

Data were described as frequencies and percentages. We used the Shapiro–Wilk test to determine that the scale scores did not conform to a normal distribution and presented data as medians with interquartile ranges (IQRs). Chi-square and Fisher's exact tests were used to compare the socio-demographic differences between those with depression vs. those without depression, those with anxiety vs. those without anxiety and those with insomnia vs. those without insomnia, respectively. A binary logistic regression analysis was established to determine the risk factors for depression, anxiety, and insomnia symptoms, and outcomes were presented as odds ratios (ORs) and 95% CIs. The significance level was *P* = 0.05 at a 2-tailed. Data were analyzed using SPSS version 22.0.

## Results

### Ophthalmic Online Consultation

There were 425 online consultations during the lockdown, with an average of 71 consultations per month, including 14 invalid consultations (3.3%) and 114 repeated consultations (26.8%). The most common reason for consultation was eye discomfort or abnormal appearance (165 visits, 38.8%), and the top three most common manifestations of ocular discomfort were red, swollen, painful eyelids (38, 23.0%); red eyes, itching, blinking or rubbing eyes (27, 16.4%); and red eyes (20, 12.1%) (see Table 1).

**Table 1:** The content and proportion of online consultation.

**Consultation Content**	**Epidemic period, No. (%)**
Abnormal secretions	11 (2.6)
Blurred or different vision	20 (4.7)
Eye discomfort or abnormal appearance	165 (38.8)
Fluttering shadows before eyes	5 (1.2)
Myopia and abnormal eye position	13 (3.1)
Ocular trauma	5 (1.2)
Eye surgery	28 (6.6)
Outpatient follow-up and further treatment	22 (5.2)
Postoperative follow-up	12 (2.8)
The other	16 (3.8)
Invalid consultations	14 (3.3)
Repeated consultations	114 (26.8)

### Socio-Demographic Characteristics

A total of 139 valid questionnaires were collected in this survey from patients who were involved in the online ophthalmic consultation. There were 105 (75.5%) females and 34 (24.5%) males. Those aged between 31 and 40 accounted for a majority of 47 (33.8%). Ethnicity was also recorded, and most patients were Han, 111 (79.9%) with minorities accounting for 28 respondents (20.1%). Most respondents had a bachelor's degree, 91 (65.5%); 40 (28.8%) were health care workers; 100 (71.9%) were staying at home; and 126 (90.6%) had used electronic products, including mobile phones and computers, for a long time. Regarding their sense of trust in and satisfaction with online consultation, 109 (78.4%) trusted the online consultation, 2 (1.4%) distrusted it, and 28 (20.1%) were unsure. More than half said they were satisfied with the online consultation (*n* = 82, 59%), 32 (23%) basically satisfied, and 25 (18%) said they were dissatisfied (see Table 2).

**Table 2:** Socio-demographic variables of participants.

**Variables**	**Participants, No. (%)**
Overall	139 (100.0)
**Gender**	
Male	34 (24.5)
Female	105 (75.5)
**Age**	
<18	5 (3.6)
18–25	27 (19.4)
26–30	27 (19.4)
31–40	47 (33.8)
41–50	24 (17.3)
51–60	5 (3.6)
>60	4 (2.9)
**Ethnic group**	
Han	111 (79.9)
Minorities	28 (20.1)
**Level of education**	
Elementary School	7 (5.0)
Junior High School	6 (4.3)
Senior High School	20 (14.4)
Bachelor	91 (65.5)
Master	10 (7.2)
Doctor	5 (3.6)
**Are you a healthcare worker?**	
Yes	40 (28.8)
No	99 (71.2)
**Current status**	
Staying at home	100 (71.9)
Working outside	399 (28.1)
**Do you use electronics for a long time?**	
Yes	126 (90.6)
No	13 (9.4)
**Sense of trust in online consultation**	
Trust	109 (78.4)
Distrust	2 (1.4)
Unsure	28 (20.1)
**Satisfaction toward online consultation**	
Satisfied	82 (59.0)
Basically satisfied	32 (23.0)
Dissatisfied	25 (18.0)

### Psychological Scale Results and Associated Factors

The median (IQR) scores on the PHQ-9 were 3 (0–6). In all, 52 (37.4%) showed symptoms of depression: mild in 35 (25.2%), moderate in 11 (7.9%), moderately severe in 5 (3.6%) and severe in 1 (0.7%). The median (IQR) GAD-7 scores were 1 (0–4). Thirty-two participants (23.0%) showed symptoms of anxiety and were divided into three groups: mild (*n* = 21, 15.1%), moderate (*n* = 5, 3.6%) and severe (*n* = 6, 4.3%). The median (IQR) ISI scores were 5 (1–9). Symptoms of insomnia were reported in 53 participants (38.1%); 41 (29.5%) reported mild insomnia, 9 (6.5%) reported moderate insomnia symptoms, and 3 (2.2%) reported severe insomnia.

Depression was found to be significantly more common in health care workers (*P* = 0.019). It was also related to their satisfaction with online consultation (*P* = 0.024). There was a tendency in those who had used electronic devices for a long time to suffer from depression (*P* = 0.085). Anxiety was also more common among participants who had used electronics for a long time (*P* = 0.038). There was no significant sociodemographic difference between those with insomnia vs. those without insomnia (see Table 3).

**Table 3:** Socio-demographic differences between those with symptoms of depression, anxiety or Insomnia vs. those without depression, anxiety or insomnia.

**Variables**	**Depression** ^**a**^			**Anxiety** ^**b**^			**Insomnia** ^**c**^		
	**Participants, No. (%)**			**Participants, No. (%)**			**Participants, No. (%)**		
	**Yes**	**No**	***P* ^d^**	**Yes**	**No**	***P***	**Yes**	**No**	***P***
Overall	52 (37.4)	87 (62.6)		32 (23.0)	107 (77.0)		53 (38.1)	86 (61.9)	
**Gender**									
Male	10 (29.4)	24 (70.6)	0.267	8 (23.5)	26 (76.5)	0.935	10 (29.4)	24 (70.6)	0.229
Female	42 (40.0)	63 (60.0)		24 (22.9)	81 (77.1)		43 (41.0)	62 (59.0)	
**Ethnic group**									
Han	41 (36.9)	70 (63.1)	0.818	23 (20.7)	88 (79.3)	0.199	45 (40.5)	66 (59.5)	0.244
Minorities	11 (39.3)	17 (60.7)		9 (32.1)	19 (67.9)		8 (28.6)	20 (71.4)	
**Are you a healthcare worker?**									
Yes	21 (52.5)	19 (47.5)	**0.019**	12 (30.0)	28 (70.0)	0.214	17 (42.5)	23 (57.5)	0.5
No	31 (31.3)	68 (68.7)		20 (20.2)	79 (79.8)		36 (36.4)	63 (63.6)	
**Current status**									
Staying at home	34 (34.0)	66 (66.0)	0.183	24 (24.0)	76 (76.0)	0.661	36 (36.0)	64 (64.0)	0.408
Working outside	18 (46.2)	21 (53.8)		8 (20.5)	31 (79.5)		17 (43.6)	22 (56.4)	
**Do you use electronics for a long time?**									
Yes	50 (39.7)	76 (60.3)	0.085	32 (25.4)	94 (74.6)	**0.038**	50 (39.7)	76 (60.3)	0.241
No	2 (15.4)	11 (84.6)		0 (0.0)	13 (100.0)		3 (23.1)	10 (76.9)	
**Age**									
<18	0 (0.0)	5 (100.0)	0.375	0 (0.0)	5 (100.0)	0.738	1 (20.0)	4 (80.0)	0.85
18–25	8 (29.6)	19 (70.4)		5 (18.5)	22 (81.5)		9 (33.3)	18 (66.7)	
26–30	13 (48.1)	14 (51.9)		6 (22.2)	21 (77.8)		12 (44.4)	15 (55.6)	
31–40	18 (38.3)	29 (61.7)		13 (27.7)	34 (72.3)		17 (36.2)	30 (63.8)	
41–50	10 (41.7)	14 (58.3)		7 (29.2)	17 (70.8)		10 (41.7)	14 (58.3)	
51–60	2 (40.0)	3 (60.0)		0 (0.0)	5 (100.0)		3 (60.0)	2 (40.0)	
>60	1 (25.0)	3 (75.0)		1 (25.0)	3 (75.0)		1 (25.0)	3 (75.0)	
**Level of education**									
Elementary School	1 (14.3)	6 (85.7)	0.231	1 (14.3)	6 (85.7)	0.694	2 (28.6)	5 (71.4)	0.231
Junior High School	1 (16.7)	5 (83.3)		1 (16.7)	5 (83.3)		0 (0.0)	6 (100.0)	
Senior High School	4 (20.0)	16 (80.0)		2 (10.0)	18 (90.0)		5 (25.0)	15 (75.0)	
Bachelor	40 (44.0)	51 (56.0)		24 (26.4)	67 (73.6)		40 (44.0)	51 (56.0)	
Master	4 (40.0)	6 (60.0)		3 (30.0)	7 (70.0)		4 (40.0)	6 (60.0)	
Doctor	2 (40.0)	3 (60.0)		1 (20.0)	4 (80.0)		2 (40.0)	3 (60.0)	
**Sense of trust in online consultation**									
Trust	38 (34.9)	71 (65.1)	0.139	24 (22.0)	85 (78.0)	0.08	39 (35.8)	70 (64.2)	0.474
Distrust	2 (100)	0 (0.0)		2 (100.0)	0 (0.0)		1 (50.0)	1 (50.0)	
Unsure	12 (42.9)	16 (57.1)		6 (21.4)	22 (78.6)	13 (46.4)	15 (53.6)		
**Satisfaction toward online consultation**									
Satisfied	24 (29.3)	58 (70.7)	**0.024**	14 (17.1)	68 (82.9)	0.133	27 (32.9)	55 (67.1)	0.237
Basically satisfied	18 (56.3)	14 (43.8)		11 (34.4)	21 (65.6)		16 (50.0)	16 (50.0)	
Dissatisfied	10 (40.0)	15 (60.0)		7 (28.0)	18 (72.0)		10 (40.0)	15 (60.0)	

*^a^Depression was defined as Patient Health Questionnaire-9 score of 5 or higher.*

*^b^Anxiety was defined as Generalized Anxiety Disorder-7 score of 5 or higher.*

*^c^Insomnia was defined as Insomnia Severity Index score of 8 or higher.*

*^d^Chi-square and Fisher's exact tests as appropriate were used to compare the prevalence of mental health symptoms in different populations*.

Furthermore, the results of binary logistic regression analysis demonstrated that health care workers were at risk for depressive symptoms (OR: 2.424; 95% CI: 1.143–5.143; *P* = 0.021) (see Table 4).

**Table 4:** Binary logistic regression analysis of risk factors associated with symptoms of depression, anxiety and insomnia.

**Variables**	**Depression** ^**a**^			**Anxiety** ^**b**^			**Insomnia** ^**c**^		
	**OR**	**95%CI**	***P*-value**	**OR**	**95%CI**	***P*-value**	**OR**	**95%CI**	***P*-value**
**Gender**									
Male	0.624	0.267–1.458	0.276	1.219	0.47–3.161	0.684	0.604	0.258–1.412	0.244
Female	1[Reference]			1[Reference]			1[Reference]		
**Are you a healthcare worker?**									
Yes	2.424	1.143–5.143	**0.021**	2.15	0.806–5.732	0.126	1.045	0.443–2.463	0.92
No	1[Reference]			1[Reference]			1[Reference]		
**Current status**									
Staying at home	0.619	0.287–1.334	0.221	1.933	0.678–5.51	0.217	0.751	0.318–1.777	0.515
Working outside	1[Reference]			1[Reference]			1[Reference]		
**Do you use electronics for a long time?**									
Yes	3.277	0.691–15.54	0.135	544,474,979.2	0	0.999	2.025	0.525–7.815	0.306
No	1[Reference]			1[Reference]			1[Reference]		

*OR, Odds ratio; CI, Confidence interval.*

*^a^Depression was defined as Patient Health Questionnaire-9 score of 5 or higher.*

*^b^Anxiety was defined as Generalized Anxiety Disorder-7 score of 5 or higher.*

*^c^Insomnia was defined as Insomnia Severity Index score of 8 or higher. The bold value indicates that the results are statistically significant*.

## Discussion

This survey revealed the overall prevalence of depression, anxiety, and insomnia symptom was 37.4, 23.0, and 38.1%, respectively. Additionally, depression was found to be significantly more common in health care workers (*P* = 0.019) and those who were basically satisfied with online consultation (*P* = 0.024). Anxiety was more common among participants who had used electronics for a long time (*P* = 0.038). Interestingly, the current study found health care work was a risk factor for depressive symptoms (odds ratio [OR]: 2.424; 95% CI: 1.143–5.143; *P* = 0.021) among patients who were involved in online consultation for ocular manifestations in the prevailing circumstances of the COVID-19 pandemic.

Under COVID-2019 lockdown circumstances, online consultation became an effective means of responding to the medical needs of non-COVID-19 patients. There were 425 online consultations during the lockdown period from February to August, with an average of 71 visits per month. The number of online consultations was relatively small, and repeated consultations (26.8%) accounted for a high proportion. Many patients consulted for the same problem several times on the same day or the next day, and some even switched doctors to discuss the same problem, mostly symptom complaints. This phenomenon can be explained by patients' desire for an immediate response and their suspicion of online consultations. Meanwhile, we speculated this behavior may be influenced by COVID-19 or it may be due to personality traits. With respect to the cascade of psychological and behavioral effects triggered by the COVID pandemic, it has been shown that the negativity of the psychological effects of the lockdown was further modulated by personality traits, alexithymia, and resilience (14) and that these effects were also correlated with behavioral wellbeing such as emotional eating (15).

According to our survey, 1.4% of the study participants distrusted online consultation; 20.1% were unsure; and 18% were dissatisfied. The COVID-19 pandemic has had an impact on their access to medical care, forcing even those who do not trust in online advice to passively choose online counseling, which may increase their concern about their health. Therefore, online counseling may affect some people's mental disorders and more effort should be made to improve the quality of online medical services. Reasons for online ophthalmology consultations were as follows: 16.4% of 165 visits were for red eyes, itchiness, blinking, or rubbing eyes; 9.1% for dryness; 9.1% for pain; and 2.4% for foreign body sensation. Interestingly, dry eye disease (symptoms including dryness, discomfort, foreign body sensation, pain, itchiness, and so on) can be associated with psychological disorders (16).

In the present study, the prevalence of depression symptom was 37.4% and the insomnia symptom was 38.1%, which were higher than that reported in previous studies. In 2020, a study that surveyed the psychological impact of the pandemic on the general public during the initial stages reported that the prevalence of depression and anxiety symptoms were 30.3 and 36.4%, respectively (17). Remarkably, the prevalence of anxiety symptom reported in this study was 23%, which was lower than reported in previous studies. A meta-analysis during the COVID-19 outbreak showed that the overall prevalence of anxiety was 33%, and a total of 41 studies measured depression and anxiety as indicators of psychological effects. Among these studies, several involved patient populations, which showed that patients with pre-existing conditions or infected by COVID-19 had a higher prevalence of depression and anxiety than health care workers and the general public (18). Our study included people presenting with ophthalmic symptoms, not infected by COVID-19, and the results showed that participants had higher rates of depression and insomnia and lower rates of anxiety than those found in the general population. One important factor that should not be overlooked is that some of the subjects in the current study had dual identities: both health care workers and online consulting patients. Moreover, patients receiving ophthalmic consultation online in the current study were prone to depressive symptoms if they were also health care workers (odds ratio [OR]: 2.424; 95% CI: 1.143–5.143; *P* = 0.021). Another factor to consider is that the limited sample size may have contributed to the results.

Females were reported to have a higher prevalence of depressive and insomnia symptoms than males, but there was no statistically significant difference observed between females and males in psychological distress across all scales (see Table 3). Overall, most studies have revealed that females are more prone to developing mental health symptoms (17–19). In the current study, age was not shown to be associated with psychological distress, which was in accordance with previous studies (20).

In the current study, depression was observed to be significantly more common in health care workers and was also identified as a risk factor for worse depressive symptoms. Additionally, depression was significantly higher in those who were basically satisfied with online consultation. Anxiety and insomnia were also reported more among health care workers, but there was no statistically significant difference between health care workers and non-healthcare workers. As a result of the pandemic, health care workers in different specialties have suffered tremendous psychological pressure, as they not only worried about being infected but also about carrying the virus and infecting their families or colleagues, which may lead to various psychological problems (21–23). Moreover, a heavy workload, wearing protective equipment such as masks and isolation suits for long hours at work, and being in a closed environment for an extended period of time without the ability to drink, eat or use the bathroom for extended periods may aggravate the negative psychological impact on health care providers. In addition, a case of COVID-19 with keratoconjunctivitis as the main symptom has been reported (24), which may increase the number of health care workers in the present study who had eye discomfort or other eye disease psychological burdens. In contrast, a prior study in Singapore suggested that nonmedical health care workers had a higher prevalence of psychological distress because of reduced formal psychological support, less first-hand information, and less training in infection control measures (25).

In the present study, participants who used electronic products for a long time reported more depression, anxiety, and insomnia symptoms than those who did not, and it was significantly associated with anxiety. As a result of the lockdown during the COVID-19 pandemic, people were likely to spend more time on electronic devices, especially young people, exercise less and remain sedentary more, which is detrimental to psychological health (26). Moreover, too much screen time also has an impact on physical health, such as visual fatigue and dry eye. A great number of studies in children and adolescents have shown that high screen time is associated with increased risk of psychological problems (27–29). Additionally, giving more attention to media coverage of the COVID-19 outbreak is associated with higher psychological distress (30). However, in our study, the majority of participants had used electronic products for a long time, but the time giving attention to COVID-19 was unknown. Regarding all these findings, it is necessary to limit screen time and promote physical activities for the mental health of young people.

Specifically, previous publications have shown that decreased vision is known to worsen mental health of eye disease patients (31–33). Among online patients, the number of return visits accounted for 21.4%, including blindness-causing eye diseases such as glaucoma and uveitis that require long-term follow-up treatment. Individuals with glaucoma are more likely to have some or severe anxiety/depression problems than those without glaucoma (34). Therefore, one possible contributing factor of psychological disorders could be vision issues and return visits for consultations about blindness-causing eye diseases.

The results of this study suggest that it is necessary to pay attention to people's mental health and sleep condition in time when public health emergency occurs. The academy of Ophthalmology could team up with the academy of Psychology to come up with strategies to support those at high risk. In the context of COVID-19, online consultation can be regarded as an effective way to solve the medical needs of some people, but it does not significantly alleviate their psychological disorders. There is a need to provide appropriate psychological support for patients who consult online, especially if they were medical staff or had high screen time.

This study has several limitations as follows. First, the sample size of the questionnaire survey was relatively small, so the results may be biased. Second, the prevalence of psychological distress was based on respondents' self-reports rather than clinical diagnosis. Third, the online consultation period spanned several months, while the survey was administered over a two-week period. A longitudinal follow-up is needed to explore the possible long-term relationship between psychological symptoms and the disease. Finally, we did not investigate whether the respondents' ocular symptoms improved, and a control group may be lacking to identify the impact of ocular symptoms on patients' psychological status. The respondents were mainly people with high education levels, so the results of this survey were more likely to reflect their psychological state.

## Conclusion

During the COVID-19 pandemic and lockdown, psychological distress was highly prevalent among ophthalmological patients consulting with their doctors online. When another public health emergency occurs, special attention should be given not only to patients' ocular symptoms but also to their mental health, and appropriate psychological support should be given, especially for those who are medical staff and those who have used electronic products for a long time. This may mean, for example, encouraging patients to participate in more outdoor activities instead of spending too much time on screen and, if necessary, referring them to a psychiatrist.

## Data Availability Statement

The original contributions presented in the study are included in the article/supplementary material, further inquiries can be directed to the corresponding author/s.

## Ethics Statement

The studies involving human participants were reviewed and approved by the Ethics Committee of Zhongshan Ophthalmic Center, Sun Yat-sen University and People's Hospital of Xinjiang Uygur Autonomous Region. Written informed consent to participate in this study was provided by the participants' legal guardian/next of kin.

## Author Contributions

LD and NG contributed to analyzing the data and drafting the manuscript. JZ designed the questionnaire and collected the data. DT and AA collected and sorted the data. CZ and ML reviewed and revised the manuscript. All authors contributed to and have approved the final manuscript.

## Funding

This work was supported by the National Natural Science Foundation of China (Grant Number: 81970808) and Guangdong Natural Science Foundation (Grant Numbers: 2019A1515011196 and 2020A1515010121).

## Conflict of Interest

The authors declare that the research was conducted in the absence of any commercial or financial relationships that could be construed as a potential conflict of interest.

## Publisher's Note

All claims expressed in this article are solely those of the authors and do not necessarily represent those of their affiliated organizations, or those of the publisher, the editors and the reviewers. Any product that may be evaluated in this article, or claim that may be made by its manufacturer, is not guaranteed or endorsed by the publisher.

## References

[1] Wang C Horby PW Hayden FG Gao GF. A novel coronavirus outbreak of global health concern. Lancet (London, England). (2020) 395:470–3. 10.1016/S0140-6736(20)30185-9 31986257 PMC7135038

[2] WHO. WHO Director-General's statement on IHR Emergency Committee on Novel Coronavirus (2019-nCoV). (2020). Available from: https://www.who.int/director-general/speeches/detail/who-director-general-s-statement-on-ihr-emergency-committee-on-novel-coronavirus-(2019-ncov) (retrieved December 23, 2020).

[3] WHO. COVID-19 Weekly Epidemiological Update. (2022). Available from https://www.who.int/publications/m/item/weekly-epidemiological-update-on-covid-19—1-february-2022 (retrieved February 3, 2022).

[4] Chan JF-W Yuan S Kok K-H To KK-W Chu H Yang J. A familial cluster of pneumonia associated with the 2019 novel coronavirus indicating person-to-person transmission: a study of a family cluster. Lancet (London, England). (2020) 395:514–23. 10.1016/S0140-6736(20)30154-9 31986261 PMC7159286

[5] Liu W Xu Y Ma D. Work-Related Mental Health Under COVID-19 Restrictions: A Mini Literature Review. Front Public Health. (2021) 9:788370. 10.3389/fpubh.2021.788370 34900925 PMC8651716

[6] Chen S Yang J Yang W Wang C Bärnighausen T. COVID-19 control in China during mass population movements at New Year. Lancet (London, England). (2020) 395:764–6. 10.1016/S0140-6736(20)30421-9 32105609 PMC7159085

[7] Wu X Chen J Yun D Yuan M Liu Z Yan P. Effectiveness of an ophthalmic hospital-based virtual service during COVID-19. Ophthalmology. (2020) 128:942–5. 10.1016/j.ophtha.2020.10.012 33069751 PMC7561601

[8] Wu X Tao S Zhang Y Zhang S Tao F. Low physical activity and high screen time can increase the risks of mental health problems and poor sleep quality among Chinese college students. PLoS ONE. (2015) 10:e0119607. 10.1371/journal.pone.0119607 25786030 PMC4364939

[9] Shi L Lu Z-A Que J-Y Huang X-L Liu L Ran M-S. Prevalence of and risk factors associated with mental health symptoms among the general population in china during the coronavirus disease 2019 pandemic. JAMA Netw Open. (2020) 3:e2014053. 10.1001/jamanetworkopen.2020.14053 32609353 PMC7330717

[10] Liu Y Chen H Zhang N Wang X Fan Q Zhang Y. Anxiety and depression symptoms of medical staff under COVID-19 epidemic in China. J Affect Disord. (2021) 278:144–8. 10.1016/j.jad.2020.09.004 32961409 PMC7475769

[11] Sun X Li Y Yu C Li L. Reliability and validity of depression scales of Chinese version: a systematic review. Zhonghua Liu Xing Bing Xue Za Zhi. (2017) 38:110–6. 10.3760/cma.j.issn.0254-6450.2017.01.021 28100388

[12] He X Li C Qian J Cui H Wu W. Reliability and validity of a generalized anxiety disorder scale in general hospital outpatients. Shanghai Jingshen Yixue. (2010) 22:200–3. 10.3969/j.issn.1002-0829.2010.04.002

[13] Bai C Ji D Chen L Li L Wang C. Reliability and validity of Insomnia Severity Index in clinical insomnia patients. Chin J Pract Nurs. (2018) 34:2182–6.

[14] Osimo SA Aiello M Gentili C Ionta S Cecchetto C. The influence of personality, resilience, and alexithymia on mental health during COVID-19 pandemic. Front Psychol. (2021) 12:630751. 10.3389/fpsyg.2021.630751 33716896 PMC7943855

[15] Cecchetto C Aiello M Gentili C Ionta S Osimo SA. Increased emotional eating during COVID-19 associated with lockdown, psychological and social distress. Appetite. (2021) 160:105122. 10.1016/j.appet.2021.105122 33453336 PMC9755826

[16] Kitazawa M Sakamoto C Yoshimura M Kawashima M Inoue S Mimura M. The relationship of dry eye disease with depression and anxiety: a naturalistic observational study. Transl Vision Sci Technol. (2018) 7:35. 10.1167/tvst.7.6.35 30619655 PMC6314109

[17] Wang C Pan R Wan X Tan Y Xu L Ho CS. Immediate psychological responses and associated factors during the initial stage of the 2019 coronavirus disease (COVID-19) epidemic among the general population in China. Int J Environ Res Public Health. (2020) 17. 10.3390/ijerph17051729 PMC7084952 32155789

[18] Luo M Guo L Yu M Jiang W Wang H. The psychological and mental impact of coronavirus disease 2019 (COVID-19) on medical staff and general public—a systematic review and meta-analysis. Psychiatry Res. (2020) 291:113190. 10.1016/j.psychres.2020.113190 32563745 PMC7276119

[19] Essangri H Sabir M Benkabbou A Majbar MA Amrani L Ghannam A. Predictive factors for impaired mental health among medical students during the early stage of the covid-19 pandemic in Morocco. Am J Trop Med Hygiene. (2020) 104:95–102. 10.4269/ajtmh.20-1302 33205748 PMC7790070

[20] Balicer RD Omer SB Barnett DJ Everly GS. Local public health workers' perceptions toward responding to an influenza pandemic. BMC Public Health. (2006) 6:99. 10.1186/1471-2458-6-99 16620372 PMC1459127

[21] Li W Yang Y Liu Z-H Zhao Y-J Zhang Q Zhang L. Progression of Mental Health Services during the COVID-19 Outbreak in China. Int J Biol Sci. (2020) 16:1732–8. 10.7150/ijbs.45120 32226291 PMC7098037

[22] Kang L Li Y Hu S Chen M Yang C Yang BX. The mental health of medical workers in Wuhan, China dealing with the 2019 novel coronavirus. Lancet Psychiatry. (2020) 7:e14. 10.1016/S2215-0366(20)30047-X 32035030 PMC7129673

[23] Bao Y Sun Y Meng S Shi J Lu L. 2019-nCoV epidemic: address mental health care to empower society. Lancet (London, England). (2020) 395:e37–8. 10.1016/S0140-6736(20)30309-3 32043982 PMC7133594

[24] Cheema M Aghazadeh H Nazarali S Ting A Hodges J McFarlane A. Keratoconjunctivitis as the initial medical presentation of the novel coronavirus disease 2019 (COVID-19). Can J Ophthalmol. (2020) 55:e125–9. 10.1016/j.jcjo.2020.03.003 32284146 PMC7124283

[25] Tan BYQ Chew NWS Lee GKH Jing M Goh Y Yeo LLL. Psychological Impact of the COVID-19 Pandemic on Health Care Workers in Singapore. Ann Intern Med. (2020) 173:317–20. 10.7326/M20-1083 32251513 PMC7143149

[26] Tremblay MS Colley RC Saunders TJ Healy GN Owen N. Physiological and health implications of a sedentary lifestyle. Appl Physiol Nutr Metab. (2010) 35:725–40. 10.1139/H10-079 21164543

[27] Zhang F Yin X Bi C Ji L Wu H Li Y. Psychological symptoms are associated with screen and exercise time: a cross-sectional study of Chinese adolescents. BMC Public Health. (2020) 20:1695. 10.1186/s12889-020-09819-7 33183262 PMC7664075

[28] Zhang J Liu M-W Yu H-J Chen Q-T Tang B-W Yuan S. Associations of health-risk behaviors with mental health among Chinese children. Psychol Health Med. (2020) 1–9. 10.1080/13548506.2020.1859559 33297726

[29] Lancet. Social media, screen time, and young people's mental health. Lancet. (2019) 393:611. 10.1016/S0140-6736(19)30358-7 30782327

[30] Hao X Zhou D Li Z Zeng G Hao N Li E. Severe psychological distress among patients with epilepsy during the COVID-19 outbreak in southwest China. Epilepsia. (2020) 61:1166–73. 10.1111/epi.16544 32353184 PMC7267575

[31] Purola PKM Nättinen JE Ojamo MUI Koskinen SVP Rissanen HA Sainio PRJ. Prevalence and 11-year incidence of common eye diseases and their relation to health-related quality of life, mental health, and visual impairment. Qual Life Res. (2021) 30:2311–27. 10.1007/s11136-021-02817-1 33755897 PMC8298234

[32] Brown RL Barrett AE. Visual impairment and quality of life among older adults: an examination of explanations for the relationship. J Gerontol B Psychol Sci Soc Sci. (2011) 66:364–73. 10.1093/geronb/gbr015 21402645

[33] Kempen GI Ballemans J Ranchor AV van Rens GH Zijlstra GA. The impact of low vision on activities of daily living, symptoms of depression, feelings of anxiety and social support in community-living older adults seeking vision rehabilitation services. Qual Life Res. (2012) 21:1405–11. 10.1007/s11136-011-0061-y 22090173 PMC3438403

[34] Jung KI Park CK. Mental Health Status and Quality of Life in Undiagnosed Glaucoma Patients: A Nationwide Population-Based Study. Medicine (Baltimore). (2016) 95:e3523. 10.1097/MD.0000000000003523 27175648 PMC4902490

